# Connectome-wide network analysis of white matter connectivity in Alzheimer's disease

**DOI:** 10.1016/j.nicl.2019.101690

**Published:** 2019-02-21

**Authors:** Chenfei Ye, Susumu Mori, Piu Chan, Ting Ma

**Affiliations:** a Department of Electronic and Information Engineering, Harbin Institute of Technology at Shenzhen, Shenzhen, Guangdong Province, China; b Peng Cheng Laboratory, Shenzhen, Guangdong, China; c The Russell H. Morgan Department of Radiology and Radiological Science, The Johns Hopkins University School of Medicine, Baltimore, MD, USA; d F.M. Kirby Research Center for Functional Brain Imaging, Kennedy Krieger Institute, Baltimore, MD, USA; e National Clinical Research Center for Geriatric Disorders, Xuanwu Hospital Capital Medical University, Beijing, China; f Department of Neurobiology, Neurology and Geriatrics, Xuanwu Hospital of Capital Medical University, Beijing Institute of Geriatrics, Beijing, China; g Clinical Center for Parkinson's Disease, Capital Medical University, Beijing, China; h Key Laboratory for Neurodegenerative Disease of the Ministry of Education, Beijing Key Laboratory for Parkinson's Disease, Parkinson Disease Center of Beijing Institute for Brain Disorders, Beijing, China; i Advanced Innovation Center for Human Brain Protection, Capital Medical University, Beijing, China

**Keywords:** Diffusion tensor imaging, Alzheimer's disease, Mild cognitive impairment, Connectome, Multivariate regression, Tractography

## Abstract

A multivariate analytical strategy may pinpoint the structural connectivity patterns associated with Alzheimer's disease (AD) pathology in connectome-wide association studies. Diffusion magnetic resonance imaging data from 161 participants including subjects with healthy controls, AD, stable and converting mild cognitive impairment, were selected for group-wise comparisons. A multivariate distance matrix regression (MDMR) analysis was performed to detect abnormality in brain structural network along with disease progression. Based on the seed regions returned by the MDMR analysis, supervised learning was applied to evaluate the disease predictive performance. Nine brain regions, including the left orbital part of superior and middle frontal gyrus, the bilateral supplementary motor area, the bilateral insula, the left hippocampus, the left putamen, and the left thalamus demonstrated extremely significant structural pattern changes along with the progression of AD. The disease classification was more efficient when based on the key connectivity related to these seed regions than when based on whole-brain structural connectivity. MDMR analysis reveals brain network reorganization caused by AD pathology. The key structural connectivity detected in this study exhibits promising distinguishing capability to predict prodromal AD patients.

## Highlights

• A multivariate analysis called MDMR was performed to discover the structural connectivity patterns associated with AD.
• Several brain regions demonstrated extremely significant structural pattern changes along with the progression of AD.
• The key structural connectivity returned by MDMR is promising to predict AD conversion from mild cognitive impairment.

## 1 Introduction

Alzheimer's disease (AD) is a leading cause of dementia, causing progressive loss of memory and cognition and affecting 46.8 million people globally (Wu et al., 2017). The amyloid cascade hypothesis (Hardy, 2006; Hardy and Higgins, 1992), one of the most prevalent explanations for AD pathological mechanism, proposes that the accumulation of amyloid-β peptides leads to neurofibrillary tangles and neuron loss. Since these pathological hallmarks can appear years before the onset of evident symptoms, it is now accepted that therapeutic interventions targeted at the preclinical or prodromal stage of AD may delay the disease progression (Bakker et al., 2015). Mild cognitive impairment (MCI) has been widely considered as a prodromal phase of AD, with an annual conversion rate to AD of about 8–15% (Mitchell and Shiri-Feshki, 2009; Ritter et al., 2015). Various neuroimaging techniques allow more accurate prediction of this conversion, indicating a great potential to evaluate AD risk at an early stage (Eskildsen et al., 2013; Liu et al., 2013; Pereira et al., 2017; Westman et al., 2011).

Unfortunately, extensive treatment strategies to removing or modify amyloid-β peptides have failed to restore the cognition of AD patients (Canter et al., 2016; Hyman et al., 2012), thus motivating researchers to explore new pathological mechanisms besides the amyloid cascade hypothesis. A growing understanding of neural circuit dysfunction in AD suggests that long-range network dysfunction plays a key role in memory loss and cognition impairment in AD patients (Canter et al., 2016; Mattson, 2004). Similar to converging evidence demonstrating the vulnerability of brain functional connectivity in AD using functional magnetic resonance imaging (fMRI) (Damoiseaux et al., 2012; Greicius et al., 2004; Griffanti et al., 2015), the brain white matter (WM) structural connectivity was also found largely disrupted in AD patients (Lo et al., 2010), such as superior longitudinal fasciculus (Xie et al., 2006), splenium of the corpus callosum (Ukmar et al., 2008) and cingulum (Fellgiebel et al., 2008). Additionally, tractography technique on diffusion MR images enables researchers to investigate individual brain structural network in vivo (Mori et al., 2009). Given that many of these studies focusing on brain structural networks have utilized seed-based or network-based approaches with nodes defined a priori, we aim to perform connectome-wide association (CWAS) studies in a data-driven manner, to avoid potentially biased assumptions in understanding of how the underlying neuroanatomy is altered due to AD.

An intuitive analytical approach in data-driven CWAS studies is mass univariate testing on an individual basis. However, this approach may not offer sufficient statistical power when every connection comprising the network is tested independently (Zalesky et al., 2010). When research of interests is associated with global network properties, two main strategies in CWAS studies have been proposed to quantify structural networks as a continuum: dimensionality reduction and graph analysis. In the first, key components can be selected from an individual network matrix by different approaches, including principle component analysis (Zhan et al., 2015) and matrix factorization (Ball et al., 2017), so that the weights of components can be discriminated across groups. In the second, high-level graph metrics, such as small-world, nodal degree, and clustering coefficient, can be derived (Alexander-Bloch et al., 2013). Previous tractography studies using graph theory have reported abnormalities among MCI and AD populations in several brain areas, including the frontal (He et al., 2009; Lo et al., 2010), temporal (Fischer et al., 2015), and posterior-medial parietal cortices (Hagmann et al., 2008). While both strategies have reported abnormalities in structural networks in AD (Lo et al., 2010), they cannot adequately reflect the underlying neuroanatomy (Bullmore and Sporns, 2009). In particular, dimensionality reduction approaches are not fully exploratory because substantial information is lost by data reduction, and graph metrics are not specific to any one graph (e.g. different graphs can have identical network centrality). In contrast, exploratory multivariate analysis can complement graph theory for more detailed characterization of connectome variations without sacrificing connectome dimension, thus showing more promising for discovering the substrate of the specific structural connectivity patterns associated with AD pathology.

In this study, we sought to examine the unique pattern alterations in WM structural connectivity networks during the progression of AD. Multivariate distance matrix regression (MDMR) proposed by (Shehzad et al., 2014) allows exploring connectivity-phenotype relationships without any a priori information or parameter selection. This data-driven statistical approach has been recently employed to examine the overall pattern of functional or grey matter structural connectivity associated with various clinical phenotypes, such as anhedonia (Sharma et al., 2017), psychosis-spectrum symptoms (Satterthwaite et al., 2015), and AD (Rasero et al., 2017b). Inspired by this statistical framework, we applied MDMR to compare WM structural connectivity patterns among cognitively normal (CN) subjects, stable MCI (sMCI), MCI converting to AD (cMCI) patients, and AD patients. The connectome reorganization in AD progression (especially in early phase) identified by MDMR may allow for effective risk prediction of AD, as well as aid clinicians to develop a precise intervention for disconnected neural circuits.

## 2 Materials and methods

### 2.1 Study cohort

The current study obtained MR images from the Alzheimer's Disease Neuroimaging Initiative (ADNI) database, where participants were recruited from over 50 institutions across U.S. and Canada. Currently, around 1500 adults have been recruited in different ADNI initiatives, ages 55 to 90 years. The follow-up duration for all participants is specified in the protocols for ADNI-1, ADNI-2 and ADNI-GO (further information in www.adni-info.org).

In this cross-sectional study, we select subjects according to the label that is available for each subject for most of the visits: CN (cognitively normal), MCI and dementia from ADNI-2 dataset. After the initial search from ADNIMerge file (http://adni.loni.usc.edu/wp-content/themes/freshnews-dev-v2/documents/bio/inst_adni_merge.pdf), 243 subjects with both 3 T MR axial DTI scans and T1 weighted (T1W) images are available. Then 29 subjects were excluded for a non-monotone diagnosis, for example converting from MCI to dementia to MCI again. 38 subjects were further excluded for those where the last visit has no diagnosis available, as these subjects are considered unclear to belong to either the cMCI or the sMCI group. 15 subjects were removed for high levels of MR artifacts due to head-motion or magnetic susceptibility distortion. Explicitly, the failed quality measures due to head-motion were detected using an automated inspection tool DTIprep (Oguz et al., 2014), and subjects with extreme distortion in regions like prefrontal lobe and inferior temporal areas on b0 images by visualization were considered to meet exclusion criteria. A final subject sample of 161 participants was analyzed, including three groups that were matched for age and sex: 46 in CN, 48 in sMCI, 27 in cMCI, and 40 in AD. All participants had provided informed written consent before recruitment and filled out questionnaires approved by the respective Institutional Review Board (IRB).

### 2.2 Image acquisition, pre-processing and tractography

MR scanning of all subjects in this study followed the ADNI acquisition protocol (http://adni.loni.usc.edu/methods/documents/mri-protocols). Diffusion weighted images from axial DTI scans and T1 weighted (T1W) images from sagittal inversion recovery-prepared spoiled gradient-echo scans were collected. DTI images were acquired with the following parameters: 59 slices with thickness of 2.7 mm with no gap between slices, repetition time/echo time = 9 s/60 ms, 256 × 256 matrix with a field of view of 35 cm, and flip angle = 90. Forty-one diffusion weighted images (b = 1000 s/mm^2^) with noncollinear directions and one volume without diffusion weighting (b = 0 s/mm^2^) were obtained.

Pre-processing of diffusion weighted images included image denoising (Veraart et al., 2016), head-motion and eddy-current correction (Andersson and Sotiropoulos, 2016), and field inhomogeneity correction (Tustison et al., 2010). All pre-processing steps were performed within MRtrix3 (www.mrtrix.org), which included scripts that interfaced with external packages such as the FSL (https://fsl.fmrib.ox.ac.uk) (Jenkinson et al., 2012). After pre-processing, 4D diffusion weighted images were applied to estimate the diffusion tensor model for each voxel by a probabilistic fiber tracking algorithm (Jones, 2008). Specifically, 10,000 seeds were randomly distributed with a brain mask with fractional anisotropy (FA) values >0.1, and streamlines that were tracked with a step size of 0.1 × voxel size along the orientation of the principle eigenvector of the fitted tensor from each seed were terminated by the default configuration in MRtrix3 (curvature threshold = 0.02, 2000 steps maximum). It has been well recognized that deterministic tractography (Mori and van Zijl, 2002) and probabilistic tractography (Jones, 2008) are two prevalent and robust approaches for DTI fiber tracking in the scientific community. The probabilistic approach was used here because it shows higher anatomical reproducibility than the deterministic approach in terms of connectivity calculation (Bonilha et al., 2015).

### 2.3 Definition of WM structural networks

Following the tractography steps, we co-registered the FA images derived from the 4D diffusion weighted images to their corresponding T1W images by affine transformation. Based on the next non-linear registration from native T1W images to the ICBM152 template, we obtained an inverse warping transformation from the Automated Anatomical Labeling (AAL) atlas (Tzourio-Mazoyer et al., 2002) to the native DTI space. Therefore 90 brain regions not including the cerebellum were defined as WM structural network nodes, following the labels of the AAL atlas.

The construction of edges within a structural network, namely the brain WM connectivity, depends on the tractography performed before (Hagmann et al., 2008). A 5% density threshold was applied to retain the strong edge (95th percentile) weights for the connectivity matrix of each subject. We further defined the edge weight by the number of connections per unit surface between the end-nodes using a correction term of edge length, as first introduced by (Hagmann et al., 2008). Here, the strength of structural connectivity is better represented as the probability of a given brain region to be connected with another, rather than the strength of the underlying physiological WM fibers in neuronal pathways. In this way, a 90 × 90 symmetric connectivity matrix was obtained for each subject.

### 2.4 Multivariate distance matrix regression

To more comprehensively characterize the altered connectivity pattern caused by AD, advanced statistical methods should be applied. Recently, the multivariate distance matrix regression proposed by (Shehzad et al., 2014) shed light on this limitation by a comprehensive survey of connectome-behaviour relationships on a group level. In brief, the connectivity patterns between subjects, rather than individual local connections, were modeled as a marker of disease progression. We describe MDMR implementation in detail below.

We applied MDMR to test the variation of distance in connectivity patterns between groups. First, a distance matrix in the subject space was calculated for each region. Within each distance matrix, the distance between connectivity patterns for every possible subject pair among all groups related to region i was calculated by

$$d_{\mathrm{uv}}^{i}=\sqrt{2\left(1-r_{\mathrm{uv}}\right)}$$

where *r*_*uv*_ is the Pearson correlation coefficient between connectivity vectors of subject *u* and *v*. Here the connectivity vector of one subject refers to the connection of the given brain region to the rest 89 regions. Next, we performed MDMR to yield a pseudo-F estimator for the cross-group analysis, by measuring the significance of between-group variation as compared to within-group variations (Shehzad et al., 2014). Specifically, the total sum of squares for region *i* was calculated by

$${\mathrm{SS}}_{T}^{i}=\frac{1}{n}\sum_{u=1}^{n}\sum_{v=u+1}^{n}{d_{\mathrm{uv}}^{i}}^{2}$$

with *n* = *n*_1_ + *n*_2_ being the total number of subjects in the case of two groups. Likewise, the within-group sum of squares was represented by

$${\mathrm{SS}}_{W}^{i}=\frac{1}{n_{1}}\sum_{u=1}^{n}\sum_{v=u+1}^{n}{d_{\mathrm{uv}}^{i}}^{2}\varepsilon_{\mathrm{uv}}^{a}+\frac{1}{n_{2}}\sum_{u=1}^{n}\sum_{v=u+1}^{n}{d_{\mathrm{uv}}^{i}}^{2}\varepsilon_{\mathrm{uv}}^{b}$$

where *n*_1_and *n*_2_ denote the number of the first and second group respectively, *ε*_*uv*_^*a*^ equals 1 if subjects *u* and *v* belong to the first group and zero otherwise, and similarly *ε*_*uv*_^*b*^ equals 1 if subjects *u* and *v* belong to the second group and zero otherwise. Given the between-group variation denoted by *SS*_*A*_^*i*^ = *SS*_*T*_^*i*^ − *SS*_*W*_^*i*^, we could calculate the pseudo-F statistic by

$$F^{i}=\left(n-1\right)\frac{{\mathrm{SS}}_{A}^{i}}{{\mathrm{SS}}_{W}^{i}}$$

**Fig. 1:**
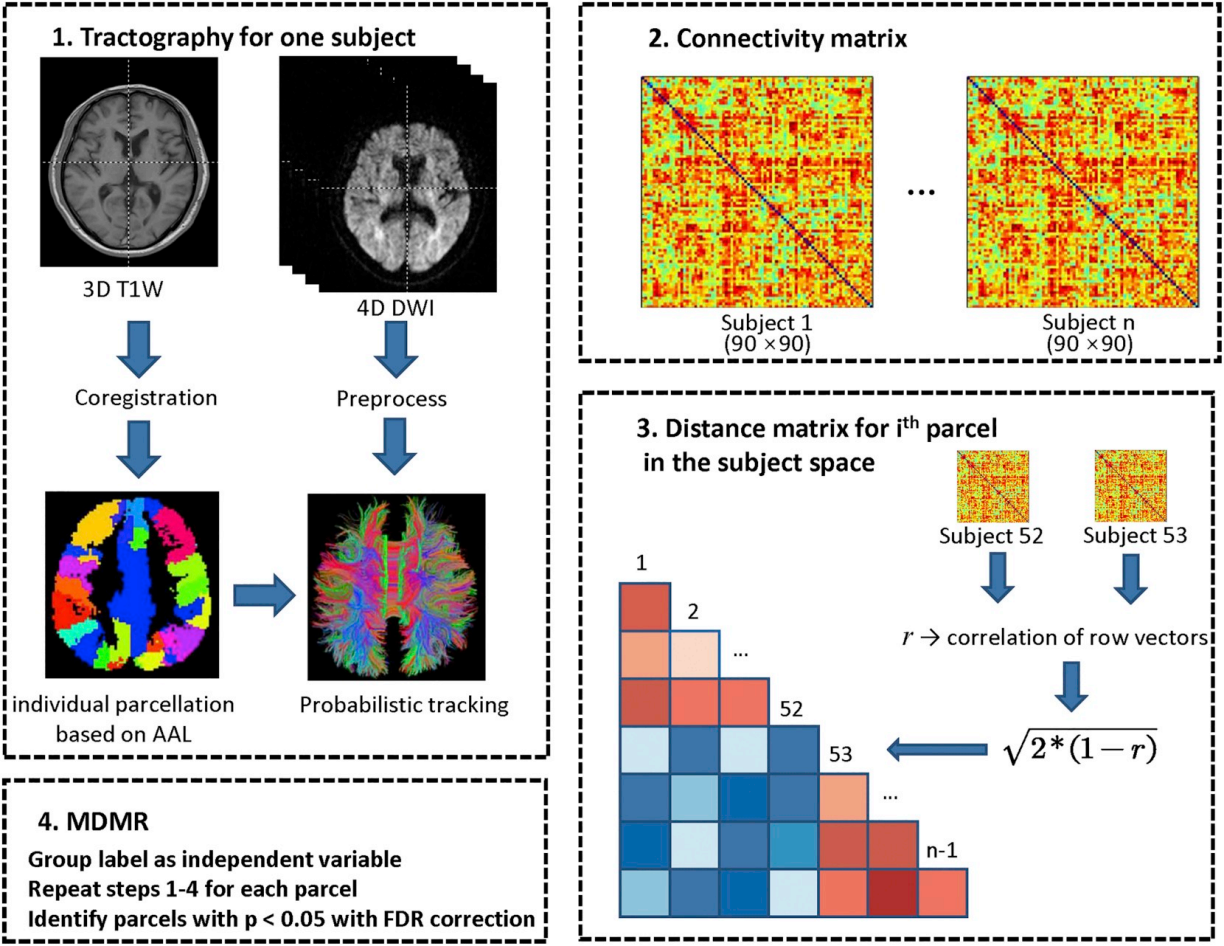
Schematic flowchart of multivariate distance matrix regression analysis based on tractography of diffusion tensor images and parcellation of 3D T1-weighted (T1W) images. DWI: Diffusion weighted imaging; AAL, automated anatomical labeling; FDR, false discovery rate.

By randomly shuffling the subject indices, a p value was calculated by counting the pseudo-F statistics from permutated values greater than those derived from the original data. Age, sex and APOE-4 level were incorporated in this model as covariates. Finally, the same procedure was repeated for *i* = 1, 2, ∙  ∙  ∙ , 90 brain regions defined in the AAL atlas. The false discovery rate correction was applied to control for type I errors due to 90 comparisons. The workflow of MDMR analysis is shown in Fig. 1. We performed MDMR for both the two-group (CN vs. sMCI, CN vs. cMCI and CN vs. AD) and three-group (CN, cMCI and AD) comparisons.

### 2.5 Post-hoc analysis

While the overall connectivity pattern of a single node of the whole-brain network associated with AD progression can be identified by MDMR, it is often a substantive interest to describe which specific connectivity pattern is primarily driving this association. Recently the δ statistic was proposed to measure the univariate effect size on a particular response variable by a randomization procedure [see (McArtor et al., 2017) for details]. For the *post-hoc* analysis, we used this statistic to identify the top five connected network nodes with the greatest effect size for each seed region returned by MDMR on the three-group comparison and examined the alteration in the connectivity pattern in those five pairwise connections for each seed region. Note that this *post-hoc* analysis subsequent to MDMR was conducted descriptively, as the seed regions were selected based on the significance (p < .001) from the MDMR results.

### 2.6 Group-wise comparison of the overall connectivity strength

To examine whether the amplitude of the structural connectivity related to local regions was altered, we compared the overall connectivity strength of each node, i.e., the sum of the absolute value over all entries of the connectivity matrix for each individual, from the same seed regions returned by MDMR analysis. A general linear model was employed to compare the overall connectivity strength between groups, using the same covariates as those listed above in MDMR analysis. A *post-ho*c permutation test was further performed to evaluate the difference between each two-group pairs. A p-value of 0.05 was set as the threshold to determine significance.

### 2.7 Classification based on structural connectivity

In order to measure the predictive performance of the key structural connectivity revealed by MDMR and *post-hoc* analysis, we further use a supervised learning algorithm to predict the cMCI and AD patients based on all the connectivity features with the greatest effect size. Partial least squares discrimination analysis (PLS-DA) was selected as the classifier here to reduce chances of overfitting. PLS-DA is the least restrictive extension of the multiple linear regression model, thus can increase the potential for generalization of the results and be of direct importance for clinical guidance. Our subject samples were randomly divided into training set (n = 129) and test set (n = 32). To reduce the variability in the classification results from the random partition of the subject samples, we repeated this procedure for five times. The structural connectivity with the greatest effect size in the *post-hoc* analysis based on the training set were selected as features for learning. The initial hyperparameters were determined using default settings in *R's caret* package, and then model tuning was performed to construct the final model. We performed binary classification for the two-group pairs (i.e. CN vs. cMCI, CN vs. AD) and five-fold cross validation within the training set to ensure generalization. Additionally, we tested the predictive performance of the full connectivity of the brain (90 × 89/2 = 4005 pairs) for comparison, with statistical inferential assessments of model performance using *diff.resamples* function in *R's caret* package (Hothorn et al., 2005). The classification performance was evaluated by sensitivity, specificity, and Receiver Operating Characteristic (ROC) curve on the test set, and Area Under Curve (AUC) of ROC was also calculated. All statistical analyses and classifications in our study were performed in R (https://www.r-project.org).

## 3 Results

### 3.1 Demographic and clinical information

Table 1 shows the demographic and clinical characteristics for CN, sMCI, cMCI, and AD subjects. No significant differences in age (p = .62), sex (p = .31), or education (p = .20) were present among subject groups. Montreal Cognitive Assessment (MoCa) scales, Rey Auditory Verbal Learning Test (RAVLT) scales and Everyday Cognition in different domains, which are cognitive functions questionnaire filled out by patients (EcogPt) significantly decreased from CN to MCI stage to AD stage (p ≤ 0.001). Cerebrospinal fluid biomarkers including Aβ_1–42_ and tau level at baseline, as well as ApoE-4 genetic phenotypes also showed significant variations among the four subject groups.

**Table 1:** Demographic and clinical information across groups.

Characteristic	CN (n = 46)	sMCI (n = 48)	cMCI (n = 27)	AD (n = 40)	Statistic	P value
Age (y)	74.5 ± 5.9	74.5 ± 8.4	76.5 ± 7.3	74.6 ± 7.7	0.62^a^	0.60
Sex (F|M)	24|22	18|30	11|16	17|23	3.60^b^	0.31
Education (y)	16.5 ± 2.8	15.8 ± 2.8	15.9 ± 2.6	15.2 ± 2.8	1.57^a^	0.20
MoCa score	26.2 ± 2.4	23.7 ± 2.7	21.4 ± 3.6	15.9 ± 5.1	68.76^a^	<0.001
RAVLT immediate recall score	45.3 ± 10.9	34.5 ± 8.8	28.3 ± 7.1	20.2 ± 7.0	63.59^a^	<0.001
EcogPt language	1.4 ± 0.4	1.9 ± 1.7	1.8 ± 0.6	1.9 ± 0.8	6.70^a^	<0.001
EcogPt visuospatial abilities	1.2 ± 0.4	1.4 ± 0.6	1.6 ± 0.7	1.9 ± 0.8	10.06^a^	<0.001
EcogPt planning	1.2 ± 0.4	1.4 ± 0.5	1.5 ± 0.6	1.9 ± 0.9	9.35^a^	<0.001
EcogPt divided attention	1.5 ± 0.5	1.9 ± 0.8	2.0 ± 0.8	2.1 ± 0.9	5.66^b^	0.001
ApoE-4 carriers (%)	0%	16.7%	22.2%	15%	23.91^b^	<0.001
Baseline Aβ_1–42_ (pg/ml)	211.0 ± 51.5	171.0 ± 54.6	148.0 ± 32.5	131.0 ± 33.6	17.90^a^	<0.001
Baseline tau (pg/ml)	62.0 ± 25.0	90.9 ± 58.6	115.0 ± 61.2	133.0 ± 55.1	10.70^a^	<0.001

CN: cognitively normal; sMCI, stable MCI; cMCI, converted mild cognitive impairment; AD, Alzheimer's disease; F, female; M, male; MoCa, Montreal Cognitive Assessment.

a F statistic obtained by using one-way analysis of variance.

b χ^2^ statistic obtained using the χ^2^ test.

### 3.2 Brain regions with altered connectivity

The results returned by MDMR analysis are included in Table 2. In brief, only one brain region (i.e. the right medial orbital part of superior frontal gyrus) was observed with significant difference in terms of connectivity patterns between the CN and sMCI groups. 13 brain regions mainly including frontal, temporal lobes, limbic areas and basal ganglia structures were observed with significantly different connectivity patterns between the CN and cMCI groups. As the disease progressed, significantly different connectivity patterns were found in 33 brain regions between the CN and AD group. The full names of all AAL brain region abbreviation are listed in Supplemental Table S1.

**Table 2:** Brain regions with significantly altered connectivity patterns returned from two-group MDMR analysis.

Brain regions	CN vs. sMCI		CN vs. cMCI		CN vs. AD	
	Pseudo-F statistic	p value (FDR corrected)	Pseudo-F statistic	p value (FDR corrected)	Pseudo-F statistic	p value (FDR corrected)
PreCG.R	1.663	0.293	1.644	0.235	2.159	0.040⁎
ORBsup.L	2.522	0.113	2.373	0.078	2.551	0.023⁎
ORBmid.L	3.677	0.054	3.629	0.048⁎	3.360	0.015⁎
IFGtriang.R	1.669	0.323	2.014	0.160	2.502	0.040⁎
ORBinf.L	1.555	0.373	4.034	0.045⁎	3.384	0.008⁎⁎
ROL.R	1.531	0.424	3.489	0.048⁎	3.748	0.012⁎
SMA.L	3.711	0.135	4.853	0.048⁎	9.149	<0.001⁎⁎⁎
SMA.R	4.275	0.054	6.184	0.030⁎	8.474	<0.001⁎⁎⁎
ORBsm.R	3.789	<0.001⁎⁎⁎	1.144	0.458	3.086	0.026⁎
INS.L	1.832	0.219	2.153	0.078	3.986	<0.001⁎⁎⁎
INS.R	1.695	0.251	2.714	0.048⁎	3.584	<0.001⁎⁎⁎
ACG.R	1.698	0.282	1.267	0.432	2.167	0.049⁎
DCG.R	2.269	0.219	2.826	0.078	2.470	0.046⁎
PCG.L	1.252	0.434	0.507	0.938	2.503	0.042⁎
PCG.R	1.384	0.424	0.910	0.677	2.528	0.040⁎
HIP.L	1.614	0.323	1.804	0.186	4.476	<0.001⁎⁎⁎
HIP.R	1.363	0.424	4.180	0.048⁎	2.674	0.056
PHG.L	1.869	0.238	1.982	0.133	2.211	0.037⁎
CAL.L	1.170	0.462	1.310	0.374	2.392	0.040⁎
CUN.L	0.803	0.749	1.181	0.432	3.920	0.008⁎⁎
SOG.L	1.553	0.323	2.379	0.078	2.240	0.048⁎
SOG.R	1.584	0.373	2.154	0.118	2.720	0.019⁎
IOG.L	2.064	0.201	1.481	0.304	2.222	0.046⁎
FFG.R	1.746	0.312	1.865	0.204	2.387	0.046⁎
SPG.R	1.227	0.434	2.425	0.078	2.704	0.015⁎
PCUN.L	0.716	0.789	1.710	0.243	4.054	0.008⁎⁎
PCUN.R	0.872	0.690	3.039	0.056	3.007	0.012⁎
CAU.R	1.894	0.294	4.228	0.030⁎	2.085	0.095
PUT.L	1.612	0.282	2.937	0.030⁎	2.669	0.012⁎
PUT.R	1.398	0.383	2.334	0.048⁎	2.071	0.066
PAL.L	1.450	0.323	2.091	0.048⁎	1.574	0.116
THA.L	2.267	0.219	2.176	0.118	4.752	<0.001⁎⁎⁎
THA.R	1.678	0.312	1.863	0.230	4.887	0.012⁎
STG.R	1.278	0.434	2.963	0.048⁎	2.379	0.039⁎
TPOsup.L	1.886	0.251	2.492	0.048⁎	2.478	0.015⁎
TPOmid.L	1.969	0.195	1.681	0.204	3.251	<0.001⁎⁎⁎
TPOmid.R	1.206	0.453	1.868	0.133	2.922	<0.001⁎⁎⁎

CN: cognitively normal; sMCI, stable MCI; cMCI, converted mild cognitive impairment; AD, Alzheimer's disease; MDMR, multivariate distance matrix regression; FDR, false discovery rate; L, left; R, right.

⁎ p < .05.

⁎⁎ p < .01.

⁎⁎⁎ p < .001.

### 3.3 Connectivity patterns for seed regions

**Fig. 2:**
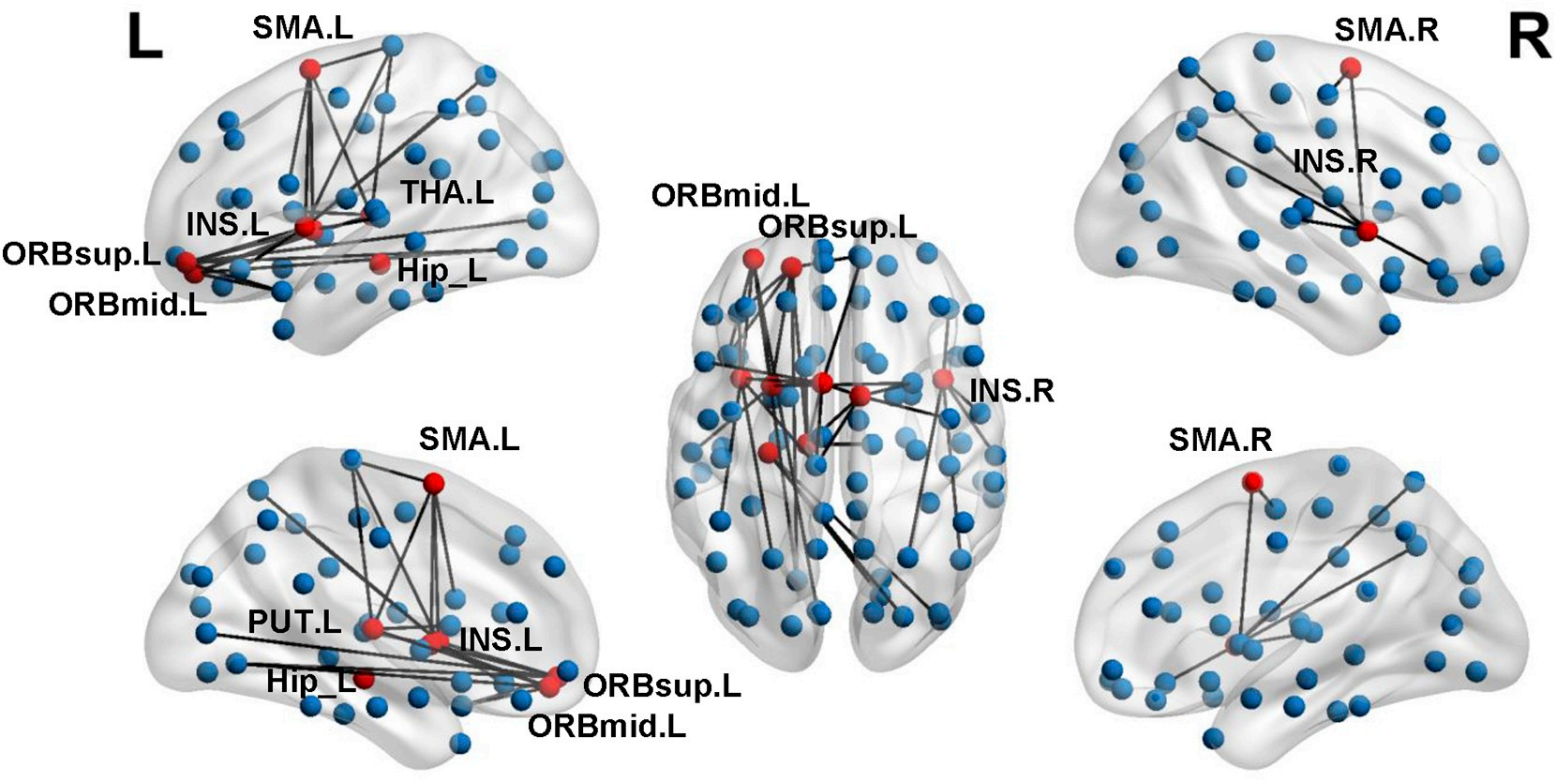
Comprehensive connectivity patterns for all seed regions identified by the post-hoc analysis. All network nodes defined in the Automated Anatomical Labeling atlas are shown, where some seed regions are represented in red and others in blue. The edges with top five greatest connectivity strength for each seed region are displayed in black. ORBsup: orbital part of superior frontal gyrus; ORBmid: orbital part of middle frontal gyrus; SMA: supplementary motor area; INS: insula; HIP: hippocampus; PUT: putamen; THA: thalamus; L, left; R, right.

**Fig. 3:**
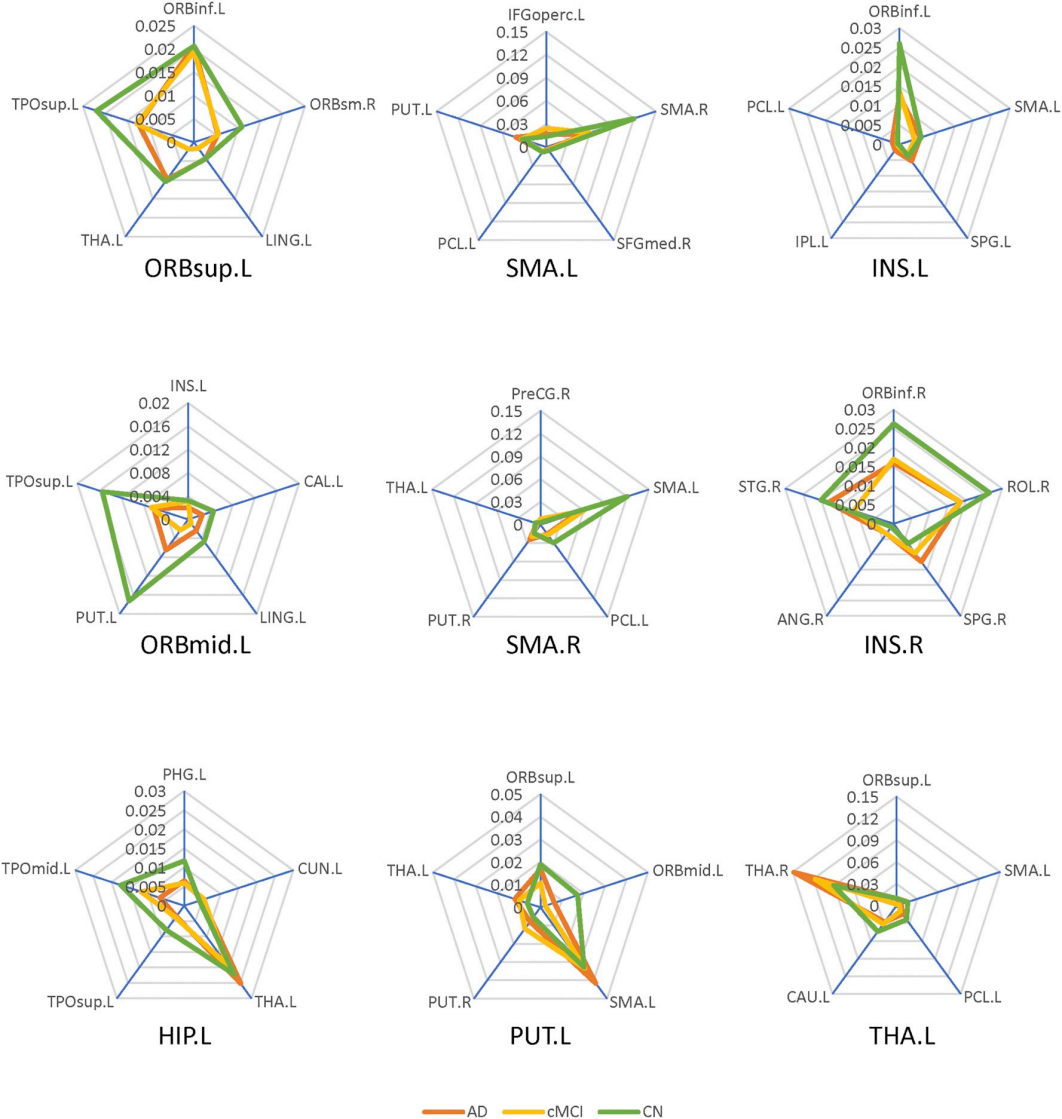
Post-hoc analysis of connectivity patterns for the seed regions subsequent to multivariate distance matrix regression (MDMR). Nine nodes with significant differences in connectivity patterns in the three-group comparison were selected for the post-hoc analysis. For each seed region returned by MDMR, the top five connections with the greatest effect size are represented as axes in the radar chart. ORBsup: orbital part of superior frontal gyrus; ORBmid: orbital part of middle frontal gyrus; SMA: supplementary motor area; INS: insula; HIP: hippocampus; PUT: putamen; THA: thalamus; L, left; R, right.

**Fig. 4:**
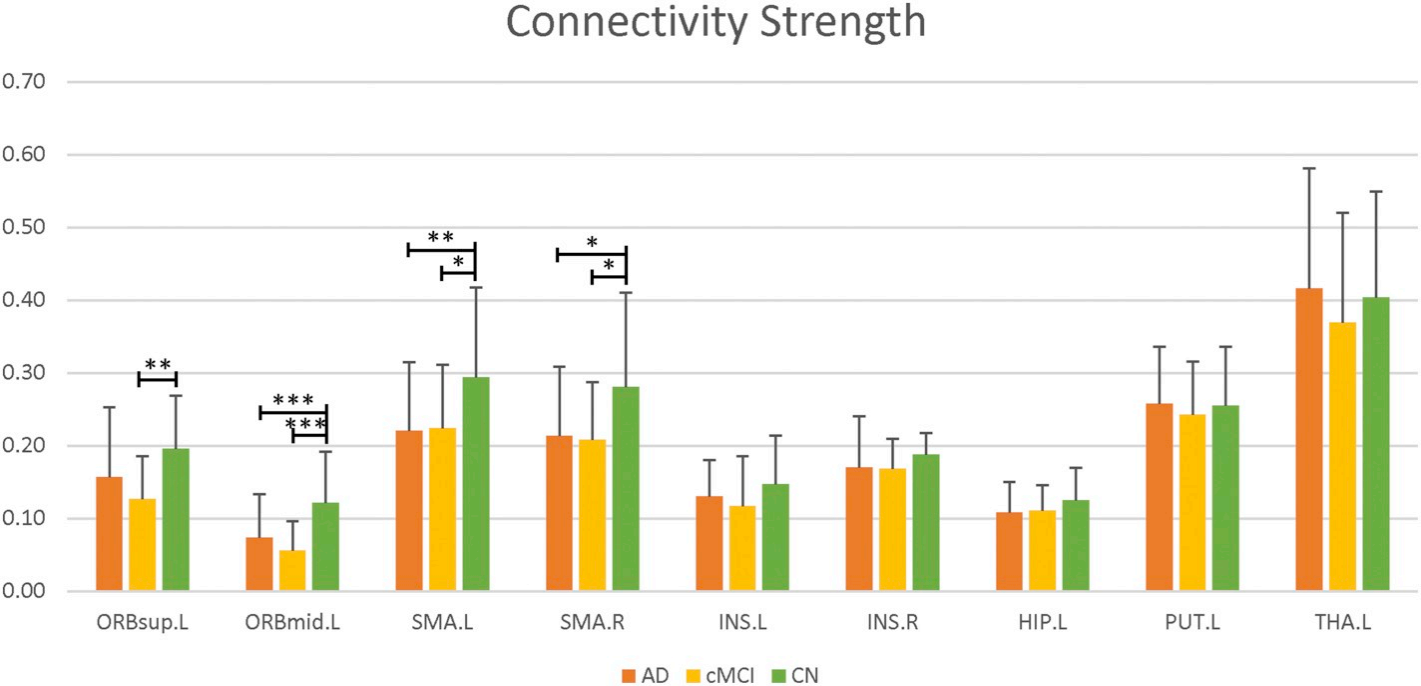
Comparison of the overall connectivity strength between groups. *, p < .05. **, p < .01. ***, p < .001. ORBsup: orbital part of superior frontal gyrus; ORBmid: orbital part of middle frontal gyrus; SMA: supplementary motor area; INS: insula; HIP: hippocampus; PUT: putamen; THA: thalamus; L, left; R, right.

From the three-group comparisons based on MDMR analysis, nine seed regions showed significant structural pattern changes were adopted as seed regions for further analysis, including the left orbital part of superior and middle frontal gyrus, the bilateral supplementary motor area, the bilateral insula, the left hippocampus, the left putamen, and the left thalamus (p < .001, Supplemental Table S2). The comprehensive connectivity patterns for all seed regions identified from the *post-hoc* analysis are qualitatively demonstrated in Fig. 2 using BrainNet Viewer toolkit (Xia et al., 2013). All network nodes defined in AAL are shown, where some seed regions are represented in red and others in blue. The edges with top five greatest connectivity strength for each seed region are displayed in black. We further applied radar charts to quantitatively characterize the connectivity patterns for those seed regions (Fig. 3), with the axes representing the connectivity strength with great effect size. The progressive decline of single structural connectivity strength along with the progression of AD was observed particularly in pairs between the left putamen and the left orbital part of middle frontal gyrus and pairs between the left hippocampus and middle temporal pole. Although the areas within the pentagon in the radar chart for the CN group appeared generally larger than in the AD and cMCI groups, the shapes at different disease stages evolved irregularly, especially for connectivity patterns relating to the left thalamus. When comparing the overall connectivity strength of the nine seed regions between groups, we found four regions showing significantly decreased amplitude in patient groups, including the left orbital part of superior frontal gyrus (p = .008 for cMCI vs. CN; Fig. 4), the left orbital part of middle frontal gyrus (p < .001 for AD vs. CN and cMCI vs. CN; Fig. 4), the left supplementary motor area (p = .007 for AD vs. CN, and p = .023 for cMCI vs. CN; Fig. 4) and the right supplementary motor area (p = .015 for AD vs. CN, and p = .032 for cMCI vs. CN; Fig. 4).

### 3.4 Classification performance

The two-group PLS-DA classification performance based on the structural connectivity (CN vs. cMCI and CN vs. AD) is demonstrated in Table 3. In brief, the prediction of cMCI individuals from the CN group exhibited the most accurate performance (AUC = 0.862 for key structural connectivity) among all combinations. Furthermore, the prediction of cMCI individuals from the CN group based on the key structural connectivity returned from MDMR analysis achieved significantly better performance than the full structural connectivity in the whole brain (p = .045 for sensitivity and p = .042 for AUC).

**Table 3:** The classification performance comparison between features from whole-brain connectivity and key connectivity from three-group MDMR analysis.

Classification measurements	CN vs. cMCI		CN vs. AD	
	whole-brain connectivity features	MDMR connectivity features	whole-brain connectivity features	MDMR connectivity features
Sensitivity	0.547	0.713⁎	0.719	0.670
Specificity	0.850	0.793	0.701	0.762
Area under ROC	0.783	0.862⁎	0.785	0.817

⁎ Classification measurements based on MDMR connectivity features are significantly higher than those based on the whole-brain connectivity features (p < .05).

## 4 Discussion

This study aimed to identify pattern changes in brain structural connectivity caused by AD pathology. For this purpose, we employed DTI tractography to establish individual-based connectivity networks in CN, sMCI, cMCI, and AD groups and applied MDMR with *post-hoc* analysis to detect network abnormalities between different phases of disease severity in a comprehensive way. The multi-variate data-driven analytical framework employed in this study included no a priori knowledge for seed nodes, while demonstrated capability to efficiently detect the local connection that mostly contributing to the abnormal topology of the brain network. This approach improves our understanding of the association between brain structural connectivity and AD progression in several ways: 1) extensive disruption of structural connectivity occurs in both prodromal and clinical stage of AD; 2) despite the contribution of amplitude changes, the connectivity pattern alteration implies potential roles for the complex circuit dysfunction underlying AD pathophysiology; and 3) several key structural disrupted connections demonstrate a promising distinguishing capability for predicting MCI individual converting to AD. Taken together, these findings delineate the extensive reorganization of brain structural connectivity across clinical diagnostic categories.

Detecting abnormal connectivity patterns of structural brain networks is a central aim of MDMR analysis. We first revealed abnormalities during the conversion from CN to cMCI stage in 13 brain regions encompassing the default mode network (DMN), including the superior temporal pole and hippocampus; the sensory-motor network, including supplementary motor areas; the orbitofrontal cortex, the insula, and the striatum. Our findings are consistent with the reported dysregulation of multiple functional networks in prodromal and clinical AD patients using fMRI techniques (Badhwar et al., 2017). Given that a strong correlation between structural and functional connectivity has been recently verified in brain networks, including the DMN, the identified structural disconnection revealed by MDMR analysis may reflect the underlying connectivity changes induced by functional disorders. In contrast to these 13 regions showing significant network pattern difference between cMCI and CN groups, only one region with significant difference was observed between sMCI and CN groups, indicating potential to distinguish different disease trajectories in early stage. Additionally, the disconnection pattern was more extensive between CN and AD subjects, highlighting the remarkable disruption of structural networks in the clinical stage of AD. The identified 33 regions, widely distributed in frontal, temporal, occipital, and limbic areas, with significant connectivity differences between CN and AD subjects, are largely consistent with those reported in a number of previous studies (Daianu et al., 2013; Lo et al., 2010; Wee et al., 2011). We speculate that these disconnection patterns may explain the worsening of several cognitive functions, including episodic memory (Tromp et al., 2015), verbal fluency (Mueller et al., 2015), executive functions (Allain et al., 2013), visuospatial skills (Vlcek and Laczo, 2014), and attention (Backman et al., 2005), which are reflected in RAVLT and EcogPt scales in Table 1.

The follow-up analysis subsequent to MDMR explicitly described which specific patterns of structural connectivity were responsible for the significant results. Notably, we found that the intrinsic connectivity patterns among the nine seed regions with structural connectivity alterations vary differently as AD pathology progresses. For the bilateral supplementary motor area and left insula, the major pattern changes caused by AD were reflected as a progressive connectivity decline along with some specific axes in the radar chart, while the connectivity strength along with other axes generally remained integral. In particular, the connectivity between the bilateral supplementary motor area in cMCI and AD subjects exhibited reduced strength, consistent with the overall connectivity decline. The overlapping connectivity abnormalities between amplitude and pattern analysis is mainly associated with the well-known impairment of movement blindness (Buchman and Bennett, 2011). Moreover, this finding may reflect the impaired underlying commissural fibers in AD progression, as supported by numerous DTI studies focusing on the disrupted integrity of the corpus callosum in MCI and AD patients (Preti et al., 2012; Preti et al., 2011; van Bruggen et al., 2012). The remarkable connectivity reduction between the insula and the orbital part of inferior frontal gyrus in the left hemisphere is congruent with previous studies reporting that the insular cortex undergoes substantial pathological changes in AD (Bonthius et al., 2005; Foundas et al., 1997; Rombouts et al., 2000). Since the insula plays a key role in multiple regulatory mechanisms, its disconnection could be associated with autonomic dysfunction, which could trigger the leading causes of death in AD patients, including cardiac failure and bronchopneumonia (Bonthius et al., 2005). Furthermore, the frontoinsular cortex, as one of the integral hubs in the salience network, is involved in attentional processing and cognitive control (Marusak et al., 2015). Thus, the observed disconnection between the left insula and the inferior frontal gyrus provides more evidence to the theory that the structural impairment of the salience network emerges in AD progression (He et al., 2014).

We also found similarly diminished connectivity between the left hippocampal formation and temporal pole. The connected brain regions with the greatest effect size contributing to pattern alterations along with AD progression were basically areas strongly connected to the hippocampus anatomically, including limbic (amygdala, parahippocampus, fusiform) and paralimbic structures (temporal pole). Regions distributed in the limbic-temporal module were also reported to show altered structural connectivity in both late MCI and AD patients (Rasero et al., 2017a). Considerable reductions in metabolic glucose (Nestor et al., 2003) and functional connectivity (Wang et al., 2007) in limbic-temporal regions also supports the reduced structural connectivity found between the left hippocampus and the temporal pole. While the mechanism underlying the occurrence of both decreased connectivity and the paradoxical slight increase within the limbic network, e.g. in the hippocampal-thalamic connectivity, remains an open question, the disruption of the Papez circuit (Aggleton et al., 2016), which is interconnected with multiple limbic regions during AD progression may explain the significant pattern changes. Therefore, we speculate that this structural reorganization of the limbic network may account for the memory deficits observed among cMCI and AD patients. In addition, the overall reduced connectivity related to the orbitofrontal cortex in cMCI and AD patients indicates an important role for this region in AD progression. This observation is largely congruent with prior studies reporting that orbitofrontal cortex can be damaged conspicuously by neurofibrillary tangle (NFT) pathology in prodromal AD (Tekin et al., 2001; Van Hoesen et al., 2000). Regarding the left thalamus, the right insula, and the left putamen, the occurrence of both decreased and paradoxically increased connectivity within each nodal network across diagnostic categories highlights the irregular connectivity patterns developing with AD progression. In contrast, the aberrantly increased connectivity between the bilateral thalamus pair, the left putamen and the supplementary motor area pair and the right insula and the superior parietal cortex pair in patients is difficult to interpret, and caution is needed to avoid ambiguity regarding the interpretability of these structural connections. Since the magnitude of structural connectivity strength can be influenced by several physiological and methodological factors (Jbabdi and Johansen-Berg, 2011; Jones et al., 2013), further investigation and validation are required to verify whether the structural connectivity reflects the underlying white matter pathway.

The individual classification in cMCI groups based on the connectivity features returned from the MDMR exhibited significantly better discriminative performance than the full whole-brain connectivity (p = .045 for sensitivity and p = .042 for AUC), suggesting the power of connectivity features identified by MDMR to characterize the prodromal AD category. These structural connectivity features and the corresponding topological alterations found in our study may yield insights into neural circuit-damaging processes before onset of evident symptoms. Although the pathological mechanism of AD remains unclear, emerging evidence from CWAS studies shift the etiological focus of AD from a single-target to an integrated outlook, which highlights the disruption of neural circuits (De Strooper and Karran, 2016) and network connectivity (Palop and Mucke, 2010). Recent studies suggest that the progressive aggregation of amyloid-β peptides and tau levels may be induced by local circuit dysfunction in brain networks, further contributing to the subsequent abnormal activity of downstream targets (Busche et al., 2015). As the amyloid plaques and NFT propagate with the aberrant structural connectivity, brain network reorganization could explain the cognitive decline. Another important finding of our study is the fair classification performance (AUC = 0.862) achieved on prodromal patients using supervised learning, which indicates the promising predictive ability of AD conversion for the individual at risk. Although the sensitivity is not remarkably high, the identified abnormal connectivity patterns during disease progression will facilitate potential interventions to alter disease trajectory or restore memory and cognition in early AD patients.

The multi-variate analytical approach performed in this study seems an idea data-driven strategy to localize the pattern changes of an individual brain region in a brain network associated with disease progression. The mass-univariate statistical models utilized in brain network studies have suffered from less reproducibility of significantly disrupted connectivity, increasing the risk of exaggerated scientific results (Poldrack et al., 2017). Furthermore, the mass-univariate statistical approaches tend to ignore concurrent contributions from all entries within a connectivity matrix (Cole et al., 2010). Therefore, the multi-variate nature of MDMR allows to overcome the drawbacks above in CWAS studies. To the best of our knowledge, only one study has investigated the AD progression in diffusion-tensor brain network consisting of 20 modules by MDMR (Rasero et al., 2017a). In contrast, our study explicitly illustrated the anatomical brain structures with altered connectivity in a finer manner, and evaluated the predictive performance of the connectivity returned from MDMR.

Several limitations of this study should be noted. First, the optimal tractography method to sensitively detect AD effects on structural networks remains a controversial issue. The two main tractography approaches (e.g. deterministic and probabilistic) have their own respective merits; the deterministic approach is advantageous for tracking long fiber tracts, while the probabilistic approach exhibits more reliable performance (Khalsa et al., 2014). A recent study comparing nine different tractography algorithms to detect network abnormalities caused by AD also indicated no universally optimal methods (Zhan et al., 2015). In this study, the probabilistic approach was selected to construct the structural networks for two reasons: 1) the probabilistic approach has been reported as more effective against fiber-crossing issues (King et al., 2009); and 2) in the deterministic approach, the structural network derived is too sparse for MDMR analysis. Second, as discussed above, the biological interpretability of structural connectivity is challenging. Novel techniques on diffusion MR data, such as pixel-based analysis, offering greater anatomical specificity, can possibly explain whether the degeneration of WM tracts in AD patients is mainly due to axonal loss or demyelination (Mito et al., 2018). Thirdly, while the motivation of this study was to distinguish structural connectivity abnormalities in cMCI and AD patients, future work is undoubtedly necessary to probe the association between network changes and specific clinical manifestations. Finally, the cross-sectional dataset with a small sample size employed in our study may bias the MDMR results. Longitudinal investigations on bigger cohorts will be helpful to validate our connectivity results.

This study stresses the value of implementing MDMR to investigate the topological properties of structural networks on an individual basis. While sMCI patients do not exhibit global connectivity alterations, an extensive reorganization of structural networks among cMCI and AD patients occurs in a number of cortico-subcortical regions. The aberrant pattern of structural connectivity is consistent with circuit dysfunction and functional connectivity damage reported in previous studies and supports current theories on brain network disruptions caused by AD. The impaired key structural connections that we identified demonstrate the promising distinguishing capability of MDMR to predict prodromal AD patients.

## Disclosures

The authors report no biomedical financial interests or potential conflicts of interest. The data contained in the manuscript being submitted have not been previously published, have not been submitted elsewhere and will not be submitted elsewhere while under consideration at Neurobiology of Aging. All authors have reviewed the contents of the manuscript being submitted, approve of its contents and validate the accuracy of the data.

## Funding

This study was supported by the National Key Research and Development Program of China (2018YFC1312000), The Basic Research Foundation Key Project Track of Shenzhen Science and Technology Program (JCYJ20160509162237418, JCYJ20170413110656460), the Basic Research Foundation of Shenzhen Science and Technology Program (JCYJ20150403161923510), Beijing Municipal Administration of Hospitals' Mission Plan (SML20150803), Beijing Municipal Science & Technology Commission (Z161100000216140, Z171100000117013) and Beijing Municipal Commission of Health and Family Planning (PXM2017_026283_000002).

## Appendix A Supplementary data

Supplementary material Image 1
