# Supplementary material for: Connectome-wide network analysis of white matter connectivity in Alzheimer's disease
Source: Neuroimage Clin. 2019 Feb 21;22:101690. doi: 10.1016/j.nicl.2019.101690 (PMC6396432; doi:10.1016/j.nicl.2019.101690)
Supplement: Supplementary file 1 — Supplementary material [file mmc1.docx]

**Supplementary Information**

Supplementary Table 1. The full names of all AAL brain region abbreviation

| Abbreviation | Regions | Abbreviation | Regions |
| --- | --- | --- | --- |
| PreCG.L | Precentral gyrus | CUN.R | Cuneus |
| PreCG.R | Precentral gyrus | LING.L | Lingual gyrus |
| SFGdor.L | Superior frontal gyrus, dorsolateral | LING.R | Lingual gyrus |
| SFGdor.R | Superior frontal gyrus, dorsolateral | SOG.L | Superior occipital gyrus |
| ORBsup.L | Superior frontal gyrus, orbital part | SOG.R | Superior occipital gyrus |
| ORBsup.R | Superior frontal gyrus, orbital part | MOG.L | Middle occipital gyrus |
| MFG.L | Middle frontal gyrus | MOG.R | Middle occipital gyrus |
| MFG.R | Middle frontal gyrus | IOG.L | Inferior occipital gyrus |
| ORBmid.L | Middle frontal gyrus, orbital part | IOG.R | Inferior occipital gyrus |
| ORBmid.R | Middle frontal gyrus, orbital part | FFG.L | Fusiform gyrus |
| IFGoperc.L | Inferior frontal gyrus, opercular part | FFG.R | Fusiform gyrus |
| IFGoperc.R | Inferior frontal gyrus, opercular part | PoCG.L | Postcentral gyrus |
| IFGtriang.L | Inferior frontal gyrus, triangular part | PoCG.R | Postcentral gyrus |
| IFGtriang.R | Inferior frontal gyrus, triangular part | SPG.L | Superior parietal gyrus |
| ORBinf.L | Inferior frontal gyrus, orbital part | SPG.R | Superior parietal gyrus |
| ORBinf.R | Inferior frontal gyrus, orbital part | IPL.L | Inferior parietal, but supramarginal and angular gyri |
| ROL.L | Rolandic operculum | IPL.R | Inferior parietal, but supramarginal and angular gyri |
| ROL.R | Rolandic operculum | SMG.L | Supramarginal gyrus |
| SMA.L | Supplementary motor area | SMG.R | Supramarginal gyrus |
| SMA.R | Supplementary motor area | ANG.L | Angular gyrus |
| OLF.L | Olfactory cortex | ANG.R | Angular gyrus |
| OLF.R | Olfactory cortex | PCUN.L | Precuneus |
| SFGmed.L | Superior frontal gyrus, medial | PCUN.R | Precuneus |
| SFGmed.R | Superior frontal gyrus, medial | PCL.L | Paracentral lobule |
| ORBsm.L | Superior frontal gyrus, medial orbital | PCL.R | Paracentral lobule |
| ORBsm.R | Superior frontal gyrus, medial orbital | CAU.L | Caudate nucleus |
| REC.L | Gyrus rectus | CAU.R | Caudate nucleus |
| REC.R | Gyrus rectus | PUT.L | Lenticular nucleus, putamen |
| INS.L | Insula | PUT.R | Lenticular nucleus, putamen |
| INS.R | Insula | PAL.L | Lenticular nucleus, pallidum |
| ACG.L | Anterior cingulate and paracingulate gyri | PAL.R | Lenticular nucleus, pallidum |
| ACG.R | Anterior cingulate and paracingulate gyri | THA.L | Thalamus |
| DCG.L | Median cingulate and paracingulate gyri | THA.R | Thalamus |
| DCG.R | Median cingulate and paracingulate gyri | HES.L | Heschl gyrus |
| PCG.L | Posterior cingulate gyrus | HES.R | Heschl gyrus |
| PCG.R | Posterior cingulate gyrus | STG.L | Superior temporal gyrus |
| HIP.L | Hippocampus | STG.R | Superior temporal gyrus |
| HIP.R | Hippocampus | TPOsup.L | Temporal pole: superior temporal gyrus |
| PHG.L | Parahippocampal gyrus | TPOsup.R | Temporal pole: superior temporal gyrus |
| PHG.R | Parahippocampal gyrus | MTG.L | Middle temporal gyrus |
| AMYG.L | Amygdala | MTG.R | Middle temporal gyrus |
| AMYG.R | Amygdala | TPOmid.L | Temporal pole: middle temporal gyrus |
| CAL.L | Calcarine fissure and surrounding cortex | TPOmid.R | Temporal pole: middle temporal gyrus |
| CAL.R | Calcarine fissure and surrounding cortex | ITG.L | Inferior temporal gyrus |
| CUN.L | Cuneus | ITG.R | Inferior temporal gyrus |

Supplementary Table 2. The brain regions with significantly altered connectivity pattern among all three groups.

| Brain regions | Pseudo-F Statistic | p value (FDR corrected) | Brain regions | Pseudo-F Statistic | p value (FDR corrected) |
| --- | --- | --- | --- | --- | --- |
| ORBsup.L | 4.240783 | <0.001*** | SOG.L | 4.070478 | 0.032* |
| ORBmid.L | 6.305674 | <0.001*** | IOG.L | 4.337236 | 0.025* |
| ORBmid.R | 4.031313 | 0.037* | FFG.L | 3.912068 | 0.038* |
| IFGtriang.R | 3.813572 | 0.043* | PCUN.L | 4.690255 | 0.023* |
| ORBinf.L | 5.231245 | 0.008** | PCUN.R | 4.20567 | 0.025* |
| ROL.R | 5.530022 | 0.011* | CAU.R | 4.287936 | 0.048* |
| SMA.L | 11.374169 | <0.001*** | PUT.L | 4.920063 | <0.001*** |
| SMA.R | 10.353896 | <0.001*** | PUT.R | 3.512602 | 0.038* |
| INS.L | 5.383894 | <0.001*** | PAL.L | 3.598402 | 0.011* |
| INS.R | 5.456183 | <0.001*** | THA.L | 5.867865 | <0.001*** |
| DCG.R | 4.686125 | 0.020* | THA.R | 5.925816 | 0.015* |
| HIP.L | 5.977142 | <0.001*** | STG.R | 4.433276 | 0.008** |
| HIP.R | 4.443328 | 0.037* | TPOsup.L | 3.967188 | 0.011* |
| CAL.L | 3.584669 | 0.043* | TPOmid.L | 4.589282 | 0.011* |
| CUN.L | 4.343077 | 0.040* | TPOmid.R | 3.944512 | 0.011* |

* p < 0.05, ** p< 0.01, *** p< 0.001
